# A Case of Undiagnosed Schizophrenia With Catatonia in a Hispanic Adolescent: The Significance of Social Determinants in the Diagnosis and the Efficacy of Risperidone and Lorazepam in Treatment

**DOI:** 10.7759/cureus.42598

**Published:** 2023-07-28

**Authors:** Sarah Tedesco, Raquel Gonzalez, Philipa Owusu-Antwi, Raymond E Robinson, Christopher Janusz

**Affiliations:** 1 Department of Psychiatry, Richmond University Medical Center, Staten Island, USA; 2 Department of Clinical Medicine, American University of Antigua, St. John's, ATG

**Keywords:** first-episode psychosis, psychiatry, child and adolescent psychiatry, social determinants of health (sdoh), culture and social determinants of health, risperidone, obsessive-schizophrenia spectrum, lorazepam challenge, adolescent catatonia, catatonia

## Abstract

Catatonia is a potentially life-threatening motor dysregulation syndrome associated with various psychiatric, medical, or developmental conditions. It is not uncommon but rarely described in the pediatric population. The timely identification of catatonia is essential as the treatment approach differs from the differential diagnoses and possible underlying conditions.

The social determinants of health are factors that may negatively impact psychological well-being, increase the risk and prevalence of mental disorders, and deteriorate the prognosis for those who already have them. The comprehension of social determinants of health is essential because it provides a deeper understanding of the complexity of societal structures and how they influence the lives of children and families. This case demonstrates how social determinants of health may contribute to misdiagnosis, delayed diagnosis, and an increase in the incidence of mental health disorders.

We present a case report on a Hispanic adolescent with first-episode catatonia in the presence of disorganized, psychotic thoughts. The patient was successfully treated with the lorazepam challenge in conjunction with Risperidone M-Tab treatment in three days. The origin of catatonia was rooted in undiagnosed schizophrenia that had worsened over a year originating from a first-episode break that questions an untreated substance-induced psychosis: the substance is unknown, as her parents had not brought her to the emergency department at that time. The demographics of this patient have also placed her at risk for a lack of access and sociocultural aspects in the delay of treatment.

Through this case report, we aim to highlight some critical points in diagnosing and managing nonmalignant catatonia in a demographically underserved minority adolescent female. This report emphasizes the need for more data about the etiology and treatment of catatonia, especially in the pediatric population.

## Introduction

Catatonia is a complex neuropsychiatric syndrome characterized by a range of motor abnormalities, including stupor, immobility, agitation, and autonomic instability. Its variations in presentation can be broadly classified as stuporous, excited, malignant, and periodic. Stuporous catatonia is characterized by psychomotor retardation, rigidity, mutism, and staring, while excited catatonia is marked by prolonged periods of psychomotor agitation. Periodic catatonia may exhibit intermittent symptom resolution, whereas malignant catatonia represents a severe and potentially life-threatening form of the condition with exacerbations of catatonic symptoms and autonomic instability [1]. In assessing pediatric catatonia, similar symptomatology to adult cases has been considered using clinical rating scales originally designed for adults, such as the Bush-Francis Catatonia Rating Scale (BFCRS) [2,3]. The BFCRS was employed to measure the progression of severity and improvement in the case presented in this article.

While catatonia in adults and children has been a subject of discussion and research, there is a notable scarcity of published literature on catatonia specifically in adolescents. This limited volume of publications hinders our understanding and identification of this condition in adolescents. Furthermore, the available literature primarily focuses on the lorazepam challenge as a treatment option for both adults and children. Three reported cases have demonstrated the successful treatment of catatonia in adolescents using lorazepam and aripiprazole.Adolescent cases include a 16-year-old male with a history of attention-deficit/hyperactivity disorder (ADHD), a 15-year-old female, and a single case report on a 36-year-old female, who were all refractory to lorazepam [4,5]. However, despite these individual case reports, the understanding of catatonia in adolescents remains limited.

Although electroconvulsive therapy has shown effectiveness in treating catatonia, patients with an underlying psychotic disorder tend to have a poor prognosis when treated with benzodiazepines compared with other patients [6,7]. The current literature does not provide a clear understanding of the benefits of adding atypical antipsychotics to the treatment regimen in cases refractory to lorazepam. Some studies suggest that risperidone may improve catatonia symptoms, while others have reported a worsening of symptoms [8,9]. Consequently, there is a crucial knowledge gap regarding the efficacy of atypical antipsychotics in catatonia, particularly in patients with an underlying diagnosis of schizophrenia.

Given the complexity of catatonia and its impact on individuals' well-being, it is essential to advance our knowledge in this area. By conducting further studies on the efficacy of atypical antipsychotics, particularly in adolescents with an underlying diagnosis of schizophrenia, we can gain valuable insights into potential treatment approaches. Addressing this knowledge gap will help clinicians develop more effective treatment strategies and improve outcomes for adolescents with catatonia.

This article focuses on a unique case of catatonia in a 17-year-old female of Hispanic descent, highlighting her clinical presentation, treatment approach, and remarkable recovery from a profound stupor, resembling a "sleeping beauty," within a brief duration of three days. Our investigation specifically explores catatonia associated with another mental disorder, taking into account the patient's history of schizophrenia. According to the Diagnostic and Statistical Manual of Mental Disorders, Fifth Edition, Text Revision (DSM-5-TR) criteria, catatonia associated with another mental disorder involves significant psychomotor disturbance along with at least three out of the following twelve symptoms: stupor, cataplexy, waxy flexibility, mutism, negativism, posturing, mannerism, stereotypy, agitation, grimacing, echolalia, and echopraxia [10]. This patient exhibited more than three of these symptoms, which adds to the uniqueness and significance of her case. By delving into this case, we aim to contribute to the understanding of catatonia and shed light on potential factors influencing its manifestation and rapid resolution.

## Case presentation

The patient is a 17-year-old female of Mexican descent attending high school, who was brought to the psychiatric emergency department by her mother for the evaluation of psychosis as requested by her school therapist. Her therapist reported her to have exhibited bizarre behavior and appeared internally preoccupied, guarded, paranoid, and unfocused.

On appearance, the patient was in no acute distress. However, she appeared unkempt. Her speech was hesitant with decreased rate, rhythm, and volume, as she moved her lips without making a sound, with long periods of silence when needing to respond to questions asked. Her mood was anxious, and her affect was flat. She was also noted to be guarded in behavior, had intense eye contact and poor concentration, and was internally preoccupied, responding to internal stimuli. She exhibited thought blocking with disorganized thoughts and paranoid delusions. The patient did not want to establish rapport as she would frequently reply, "I don't know," and would repeat the writer's questions aloud before answering. She admitted to feeling distracted lately and not feeling safe at school. She also added that her school counselor told her that she needed to come to the hospital, but she did not know exactly why. She denied suicidal and homicidal ideations. Based on her vocabulary and educational background, the patient's intellectual function appeared to be slightly below average. Insight was impaired, and judgment was poor.

**Figure 1:**
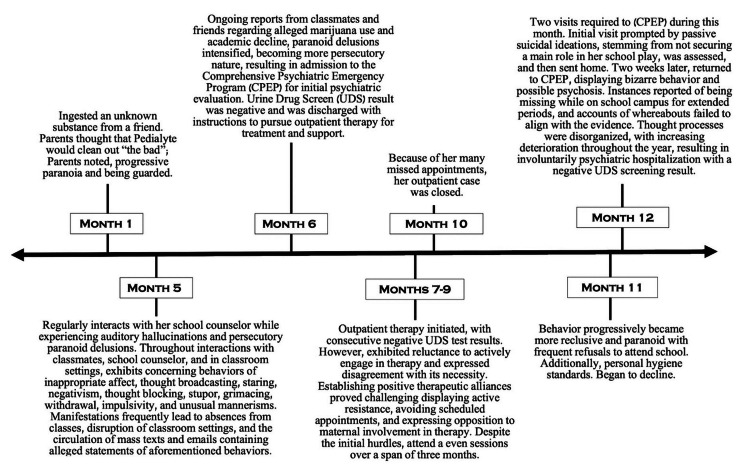
Events Leading to Admission Figure 1 illustrates the chronological timeline of the events, which had been reported to have occurred over the course of a year prior to her hospital admission

The patient's mother was Spanish native speaking, so appropriate translator services were utilized. Her mother stated that the patient had been found talking to herself, not making sense, and unfocused. She was also seen laughing and crying right after taking showers for two hours in which she scrubbed her skin until it turned red. The patient was functioning well until about a year before her current presentation to the psychiatric emergency room. She has no past medical history, and prior to the events that occurred over the course of a year, leading to her current presentation, she had no psychiatric history. It was noted that this was her first admission; however, she had visited the psychiatric emergency room a total of three times prior over the course of a year (Figure 1). Utilizing the biopsychosocial model, the patient's history was obtained by the patient's mother and her high school counselor and gathered from chart review through the electronic medical record, as the patient was not cooperative and displayed alogia (Table 1).

**Table 1:** Biopsychosocial Formulation Table 1 presents background information gathered from collateral and a thorough chart review from an electronic medical record CPEP: Comprehensive Psychiatric Emergency Program

4P Factor Model	Biopsychosocial Formulation		
	Biological	Psychological	Social
Predisposing	Family history of hypothyroidism and diabetes mellitus; individualized education plan for learning disability; menarche at 12 years of age	Impulsivity; poor problem-solving; parental conflict in mental health (stigma in not believing in mental health); and low self-esteem	Poor friendships; reports made by her friends and family for reported marijuana and nicotine use; low socioeconomic status; financial strain
Precipitating	Ongoing reported abuse of marijuana and nicotine	Stress from failing her junior year classes; not making the main role in high school play (only passing theater and English classes); interpersonal conflicts and separation with friends: thought broadcasting and auditory hallucinations; impulsivity, disrupting classes from disorganized behavior; reported substance abuse with nicotine and marijuana	School stressors, learning disability; failing classes in her junior year; not making the role in the school play
Perpetuating	Continuation of learning disability and reported abuse of marijuana and nicotine	Continuation of psychological and social predisposing factors; continuation and reinforcement of poor problem-solving; the lack of healthy intervention for the patient by the family for mental health; paranoia progression into persecutory paranoid delusions; thought blocking; progressive reclusiveness; religiosity	Role of stigma to access treatment and mental health; low socioeconomic status; poor communication among healthcare providers; poor parent-child communication; social isolation; mistrust of helping professionals; academic difficulties
Protective	Met all developmental milestones; uncomplicated pregnancy and birth; Urine drug screenings negative when tested; no past psychiatric history for the patient and family; not on any medications	Access to outpatient therapy sessions when attended after the second CPEP visit; access to the high school counselor	Stable home life with parents and two older brothers; a strong religious community of catholic faith; parental financial support; no legal history

A thorough medical workup was done, including imaging that was unremarkable and negative urine toxicology results. Other laboratory tests, including serial blood counts, metabolic panels, and thyroid functioning tests, were also normal. The parents disagreed about the patient's medication management at this time, but it was encouraging when the parents agreed to begin a second-generation antipsychotic for the patient's mood and psychosis presentation. Differential diagnosis on admission was schizophreniform disorder, substance-induced psychosis with axis I ruling out schizophrenia, major depressive disorder with psychotic features, bipolar disorder with psychotic features, and substance-induced mood disorder.

Because the patient had poor insight and judgment with poor impulse control, she was deemed a threat to herself and others, requiring psychiatric admission. Her mother agreed with voluntary hospitalization and starting medication if needed. On the contrary, her father thought it was more of a "discipline problem" and did not agree with hospitalization or medication at the time. The patient was admitted to the child and adolescent psychiatric unit. Vital signs included a heart rate of 94/minute and a blood pressure of 126/84 mmHg. Her tachycardia was attributed to dehydration, but the etiology of hypertension was uncertain at the time.

At the time of admission, she was not taking any medications. She was internally preoccupied and spent most of the day sitting fully clothed on the toilet, intensely glaring at the wall. When her mother came for the initial visit, the patient grew agitated, screaming and yelling, refusing to interact with her mother, and demanding her to leave. Shortly following the encounter, she ran into her room. When the nursing staff went to check on the patient, she was noted to be having a panic attack. She was found hyperventilating, sweating, feeling short of breath, and trembling with lightheadedness. After providing support to the patient, the patient refused to cooperate, demanded to be left alone, refused to answer any questions, and returned to the bathroom. Several hours later, during rounds, the patient was found by the nursing staff on the bathroom floor, in a posturing and waxy manner, and was repositioned to her bed by the hospital staff.

By the third day of admission, the patient deteriorated into a severe nonmalignant catatonic state; she was found on the bathroom floor, incontinent. The staff repositioned the patient from the floor to her bed. On her initial presentation, the patient scored 33 on the Bush-Francis Catatonia Rating Scale (BFCRS) for catatonia, with symptoms including immobility, staring, stupor, mutism, posturing, grimacing, negativism, waxy flexibility, withdrawal, gegenhalten, ambitendency, grasp reflex, and autonomic abnormalities. She was uncooperative, unresponsive, and lying supine in bed. Her parents were informed about her condition, consenting to emergent treatments. She also voiced concerns related to the sociocultural stigma regarding "crazy people" as the reason why consent to treatment was difficult to obtain upon admission. Since she had not taken any medications since admission, she was first started on a trial of lorazepam and Risperidone M-Tab. The patient was given intramuscular injections starting with a total dose of 3 mg of lorazepam and a total of 2 mg Risperidone M-Tab by mouth sublingually within the first 24 hours.

She grew diaphoretic with low-grade fever and was tachycardic and hypotensive. The patient had endured a decreased oral intake, causing her to suffer from dehydration and hypoglycemia, for which she was provided hydration through a syringe along with an oral glucose gel. She was also incontinent. Pediatrics was consulted, leading to recommendations including manually feeding the patient and monitoring for refeeding syndrome, monitoring fingersticks, and continuing the current treatment plan.

**Figure 2:**
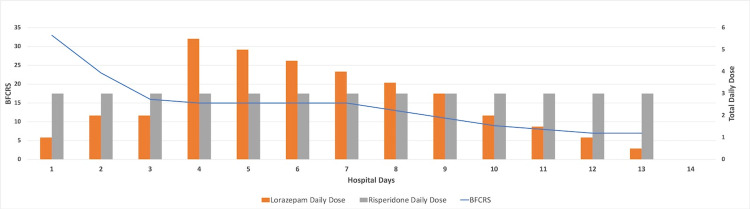
Bush-Francis Catatonia Rating Scale (BFCRS) and Medication Administration Timeline "Total Daily Dose" is noted in milligrams. "Hospital Days" reflect on the initial onset of the lorazepam challenge when the patient was noted to be found in a severe nonmalignant catatonic state

**Figure 3:**
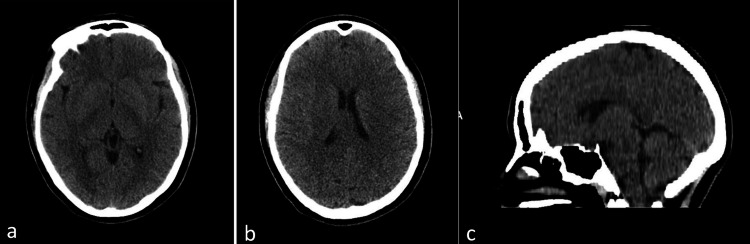
Computed tomography (CT) of the Head Without Contrast Radiology report impression: no evidence of acute intracranial process. (a) Axial/transverse view, (b) axial/transverse view, and (c) sagittal view

By day 2 of the lorazepam challenge (Figure 2), no change had been noted in the patient's catatonic state, with a continued Bush-Francis Catatonia Rating Scale score of 33, so an additional 1 mg of lorazepam was added to the patient's evening dose with a total of 4 mg lorazepam and 2 mg Risperidone M-Tab. A computed tomography (CT) scan of the head was ordered, and the findings were unremarkable (Figure 3). There was no evidence of any acute intracranial process. An EEG was also ordered, and the results were typical of a normal EEG (awake and drowsy). The nursing staff continued with assisted feeds using Ensure and water through a syringe, for adequate nutrition. The patient continued to be incontinent. See Figure 2 for the time course of medications administered and correlating BFCRS scores.

By day 3, the patient was awake, alert, and able to ambulate out of bed with assistance from the nursing staff (Figure 2). At that time, the lorazepam was transitioned from intramuscular to an oral formulation at 6 mg/day. Her Bush-Francis Catatonia Rating Scale score was 23. Because she continued to display withdrawal, the nursing staff would remind her to eat and continue to assist her. Her improvements in catatonia were sustained, and she was further monitored for any underlying symptoms of schizophrenia. During this time, she displayed episodes of paranoia, auditory hallucinations, stereotypy, echopraxia, echolalia, perseveration, and verbigeration. She continued to also display spontaneous episodes of impulsivity, negativism, staring, grimacing, mutism, rigidity, waxy flexibility, posturing, and mannerisms.

By day 14, since her onset of catatonia, she was discharged from the hospital on a medication regimen of risperidone 2 mg by mouth twice daily. At the time of discharge, her diagnosis was schizophrenia and catatonia. Her screening at discharge was at a 7 because she remained hesitant in responses and appeared to continue to persevere, overtaking her medications and wanting to feel normal. Some negativism was present, which suggested a lack of insight into her diagnosis.

She transitioned to an intensive outpatient program for those who are first-time-diagnosed schizophrenics, which helped the patient and her family cope with her diagnoses. She was also placed in outpatient therapy that occurred weekly for two weeks and then transitioned to biweekly (Table 2). She has been compliant with her scheduled outpatient appointments since discharge, and although she has some residual noted catatonic-like symptoms, as Table 2 exhibits, they remained minimal to none without any relapse in severity. She successfully returned to school one month following discharge and extracurricular activities. She continues on risperidone 2 mg/day without any signs or symptoms of catatonia.

**Table 2:** Outpatient Notes Following Discharge

Months	Medication	Catatonia Symptoms	Progress
Month 1			
First appointment	Risperidone 2.5 mg/day	Mild mutism noted as responses were slow, monotonous, and minimal	Demonstrated accurate responses to inquiries, effectively addressing questions; however, encountered difficulty sustaining the conversation beyond the initial interaction. Exhibited guarded demeanor and apathetic attitude, which constrained the depth of understanding regarding mental illness. Notably, motor activity no longer exhibited signs of psychomotor slowing
Second appointment	Risperidone 3 mg/day	Mild rigid motor activity. Constricted affect. Spontaneous speech is hesitant	Notable enhancement in academic performance reported since the previous appointment. Seems to care about her looks, noted to be wearing makeup. Continues to display flat affect and emotional constriction, but there is a discernible improvement compared to the initial session. Previously reported side effects reported to resolve. Referred to the comprehensive OnTrackNY program, which specializes in addressing the needs of individuals with schizophrenia
Month 2			
First appointment	Risperidone 3 mg/day	Exhibits guarded demeanor, displaying limited openness in communication. Motor activity appears constricted, characterized by reduced mobility and responsiveness. Noticeable decrease in speech rate and latency, reflecting challenges in verbal expression. Continues to struggle with establishing meaningful connections with peers, indicating ongoing difficulties in forming appropriate relationships	Presents as pleasant; displays a superficial level of cooperation during the interaction. Noticeable sense of motivation as evidenced by reduced response delays and congruence between her reported mood and affect. Describes current mood as "good," and expresses optimism and hope regarding involvement in the program, showcasing a positive outlook for the future
Second appointment	Risperidone 3 mg/day	No change from previous visit	Her affect was guarded at times but more euthymic than the previous session. She reports a "great" mood
Third appointment	Risperidone 3 mg/day	Average higher cognitive functions. Constricted affect	Actively engages in conversation, demonstrating improved and appropriate eye contact. Mood appeared euthymic, reflecting a balanced emotional state, while speech is less hesitant and exhibiting a more natural flow, characterized by a regular rate and latency. Notably, displayed a greater understanding and insight into her mental illness
Month 3			
First appointment	Risperidone 3 mg/day	No change from previous visit	Affect displayed moments of being guarded; it was predominantly euthymic throughout the session. However, it is important to note that limited insight into comprehending her diagnosis was still demonstrated
Second appointment	Risperidone 3 mg/day	Constricted affect	Pleasant and engaged. Somewhat guarded affect but euthymic overall
Third appointment	Risperidone 3 mg/day	Average cognitive functions	Congruent mood. Intact judgment
Month 4			
First appointment	Risperidone 3 mg/day	Somewhat guarded affect at times but euthymic overall. Social interaction is limited	Compared to previous sessions, exhibited increased proficiency in identifying and articulating emotions, and displayed a greater openness and expressiveness in communication
Second appointment	Risperidone 3 mg/day	Average motor, cognitive, and expressive development. Average social skills	Successfully forming and maintaining a friendship at school. Additionally, expresses a belief of doing well, and does not perceive any current difficulties or problems
Third appointment	Risperidone 2 mg/day	Flat affect. Incongruent mood. Difficulty expressing language. Fair memory	Affect displayed was much brighter than previous sessions
Fourth appointment	Risperidone 2 mg/day	Flat affect. Incongruent mood. Decreased speech rate	Demonstrated increased acceptance of diagnosis, and displayed ongoing willingness to explore emotions. Notably, the severity of flat and constricted affect had diminished

## Discussion

Currently, the clinical evidence supporting the use of antipsychotics for catatonia is limited, indicating only some potential benefits [1]. However, there is a lack of sufficient scientific data, particularly when it comes to the child and adolescent population. Further research is needed to establish the effectiveness and safety of antipsychotics in treating catatonia in this specific age group.

On the other hand, the lorazepam challenge has shown success in treating both adults and children with catatonia. However, it is important to note that the available data on this topic is also quite limited [11,12]. Additional studies are required to provide more comprehensive and robust evidence regarding the dosage guidelines of the lorazepam challenge in the treatment of catatonia.

In summary, while there are indications of the potential benefits of antipsychotics for catatonia, the clinical evidence is currently sparse. Further research is needed, particularly in the child and adolescent population, to better understand the effectiveness and safety of antipsychotics. Similarly, although the lorazepam challenge has demonstrated success, additional studies are necessary to establish its optimal use and efficacy in treating catatonia.

While there is a strong four-month follow-up with the patient's outpatient care, long-term monitoring of the effects of risperidone management is necessary for post-catatonic symptoms. Additionally, the chronological timeline of the onset of psychosis and the period without treatment remains unclear, which justifies the utilization of the lorazepam (Ativan) challenge in conjunction with the Risperidone M-Tab. Despite these noted variables, the patient seemed to improve right after the administration of intramuscular lorazepam. After 48 hours of being in a full-blown catatonic state, the administration of Ativan and Risperidone M-Tab helped the patient change positions in bed and increase her oral intake. A clinical study found that the deltoid intramuscular injection of lorazepam leads to the rapid absorption of the drug, with close to 100% absorption from the injection site compared to oral administration. Although there is a lag time before absorption, the first-order absorption proceeds with an average half-life of 29 minutes, which is slower than its intramuscular counterpart [13]. These results could explain the rapid response to treatment in this patient's catatonic state.

Social determinants of mental health

**Figure 4:**
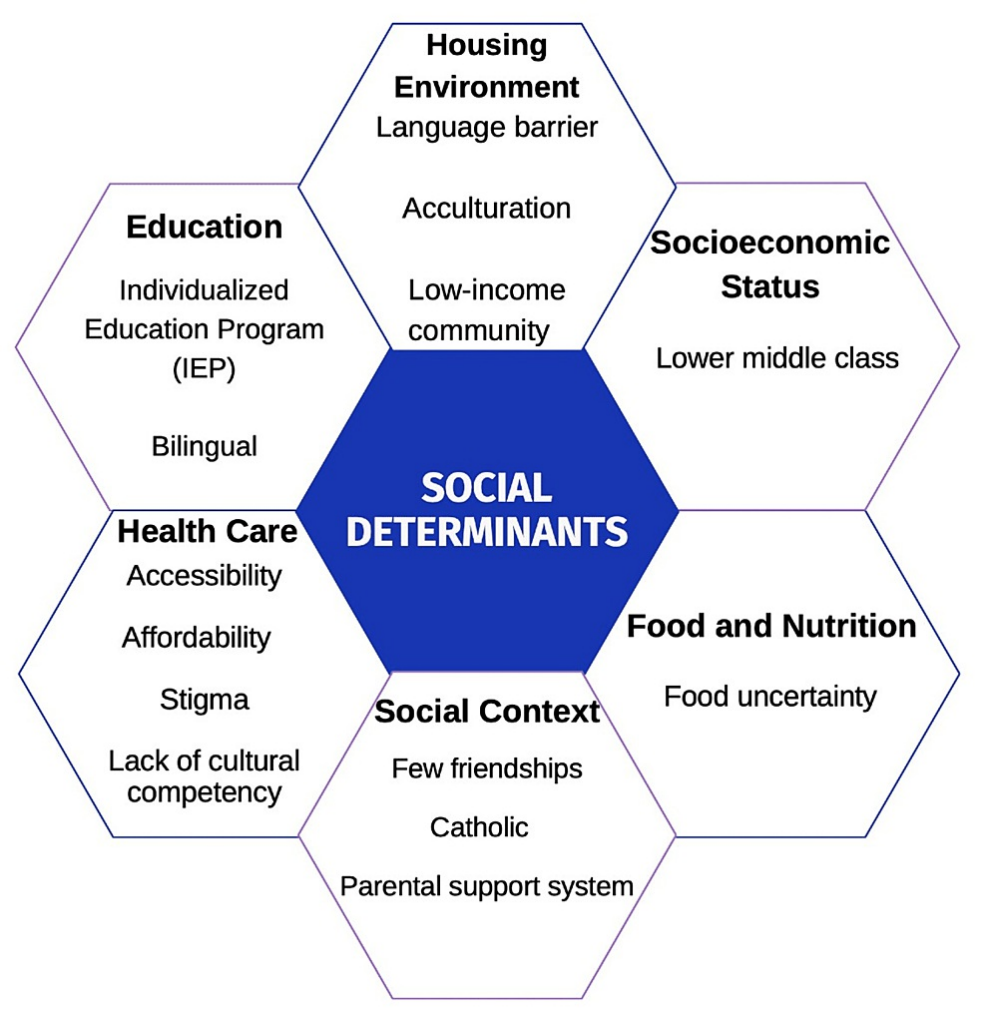
Social Determinants of Mental Health This figure portrays the social determinants of health that were exhibited in this case

To reduce discrimination and social exclusion, particularly those based on race and ethnicity, blatantly prejudiced societal norms and stigmatization must be supplanted by acceptance and social inclusion attitudes. Psychiatrists and other mental health professionals are skilled at identifying the biological, social, and psychological factors influencing their patient population. They are adept at identifying risk, predisposing, and precipitating factors that cause or exacerbate conditions using biopsychosocial and other models. In this presentation, we discuss the biopsychosocial formulation of this patient and how it relates to social determinants of mental health (Table 1 and Figure 4). It is frequently too late when a mental health professional intervenes at the risk factor level [14]. Experts in the field have advocated for moving interventions upstream, and this case study illustrates its significance.

Abuse in childhood, urban environments with a lower socioeconomic status, and substance use, particularly marijuana, have proven to be significant risk factors for increased schizophrenia spectrum disorders and poorer health outcomes [15]. The consumption of marijuana has been linked to an increased risk for an earlier onset of psychotic disorders (such as schizophrenia) in individuals with other risk factors, such as family history and genetic predisposition [16]. Numerous factors, including the quantity of drug consumed, the frequency of use, the potency and type of cannabis product, and the age of first use, have been demonstrated to impact the association between cannabis use and mental health [16].

According to the National Alliance on Mental Illness (2023), 17.0% of Hispanic/Latinx individuals live in poverty, compared to 8.2% of non-Hispanic whites. Only 35.1% of Hispanic/Latinx adults with mental illness receive annual treatment, compared to the national average of 46.2%. The Kaiser Family Foundation research in 2019 showed that 20.0% of non-senior Hispanics were uninsured [17]. In addition to a limited provider pool due to language barriers, Hispanic/Latinx uninsured individuals have even fewer options. In this case, the patient comes from a lower socioeconomic background, identifies as a minority (Hispanic female), is bilingual, and has a history and current use of unidentified substances and suspected use of marijuana. Some literature has shown that cannabis use disorder disproportionately affects minorities and adolescents [15]. According to the Substance Abuse and Mental Health Services Administration (SAMHSA), marijuana is one of the most commonly used substances among Hispanics. In 2019, marijuana use among Hispanic adolescents aged 12-17 increased significantly [18]. Other literature says more than 50% of Hispanic adolescents and young adults between the ages of 18 and 25 with severe mental illness may not receive treatment [18]. As mental illnesses frequently exacerbate without treatment, these communities are at a greater risk of developing more severe and persistent mental health conditions. Mental health providers may misunderstand or misdiagnose Hispanic/Latinx patients due to cultural differences or a lack of cultural competence [17].

In this patient's case, there was inadequate family support due to language and cultural barriers to understanding and identifying triggers and willingness to accept resources. This manifested in collateral information for a possible autism spectrum disorder diagnosis and the use of an unknown substance, which was never evaluated and further investigated, delaying necessary interventions, diagnosis, and treatment. It was only noted in the collateral that the patient had an individualized education program (IEP), not knowing the specifics of her reported learning disability. Interventions must reduce a child's exposure to abuse, neglect, substance use, and dysfunctional households to reduce future risk and assess modifiable and nonmodifiable demographic, cultural, and socioeconomic factors [19]. In addition to a clinical strategy, it is essential to implement a public health approach. Marijuana and other substance use, as well as social and demographical constructs aimed at this patient's community, may have curtailed the use of marijuana and other substances, cultural barriers, and socioeconomic factors that may have induced or worsened her onset of psychosis. An approach to public health that addresses the accessibility and affordability of resources, education, and care is essential for closing care inequities. On the communal and societal levels, psychiatrists can significantly influence policy formulation and modify social norms and stigmas. These strategies and actions will ultimately improve psychological health; reduce the incidence of mental disorders, misdiagnoses, and delayed diagnoses; and enhance patient outcomes and prognoses [12,19].

## Conclusions

This patient is the first to be noted in the literature to have been treated for catatonia successfully with the lorazepam challenge in conjunction with Risperidone M-Tab treatment with the noted origin of catatonia rooted from undiagnosed schizophrenia that had worsened over the course of a year after a first episode of psychosis. The case highlights the importance of considering social determinants of health and access to care in diagnosing and managing catatonia, particularly in demographically underserved populations. The report also emphasizes the need for more research on the etiology and treatment of catatonia, especially in pediatric patients.
